# Supplementary material for: Cells of the human intestinal tract mapped across space and time
Source: Nature. 2021 Sep 8;597(7875):250–5. doi: 10.1038/s41586-021-03852-1 (PMC8426186; doi:10.1038/s41586-021-03852-1)
Supplement: Supplementary file 1 — This file contains Supplementary Notes 1–4 and Supplementary References [file 41586_2021_3852_MOESM1_ESM.pdf]

nature portfolio

https://doi.org/10.1038/s41586-021-03852-1

Supplementary information

Cells of the human intestinal tract mapped across space and time

In the format provided by the authors and unedited

Supplementary Note 1

Poission linear mixed model for cell type composition analysis

Log linear model for two-way tables

Let Y_{ij} be the cell type count observed from the sample i ($i = 1, \ldots, N$) for the cell type j ($j = 1, \ldots, J$). A simple test of independence between samples and cell types (to make sure there is no differential cell type abundance among samples) would be to fit a log-linear model for two-way tables [1]:

$$Y_{ij} \overset{i.i.d.}{\sim} \text{Pois}(\lambda_{ij}),$$
$$\log \lambda_{ij} = \mu + a_i + b_j + \varepsilon_{ij},$$

for $i = 1, \ldots, N$ and $j = 1, \ldots, J$. Here we assume Y_{ij} follows a Poisson distribution with a mean λ_{ij}, the logarithm of which can be decomposed into the grand mean μ, the sample mean a_i, the cell type mean b_j and the interaction term ε_{ij} (between sample i and cell type j). In order to assess the two-way table is independent, we assume $\{a_i, b_j, \varepsilon_{ij}\}$ follow the independent normal distributions with variance parameters $\{\nu^2, \omega^2, \sigma^2\}$, such that

$$a_i \overset{i.i.d.}{\sim} \mathcal{N}(0, \nu^2), \quad b_j \overset{i.i.d.}{\sim} \mathcal{N}(0, \omega^2), \quad \varepsilon_{ij} \overset{i.i.d.}{\sim} \mathcal{N}(0, \sigma^2),$$

for $i = 1, \ldots, N$ and $j = 1, \ldots, J$, where the variance σ^2 is the parameter of interest. If there is no interaction (*i.e.*, no differential cell type abundance among samples), the variance estimate should become $\hat{\sigma}^2 \to 0$.

Variance explained by sample metadata

Suppose $\sigma^2 > 0$, this model enables us to explore the relative importance of a wide range of clinical/technical factors in determining cell type composition. Let x_{ik} be a value of the factor k ($k = 1, \ldots, K$) for the sample i, which is either a numerical value (*e.g.* patient's age) or a categorical value of L_k levels (*e.g.*, disease severity with $L_k = 6$: healthy, asymptomatic, mild, moderate, severe and critical). Then the mean of the poisson distribution can be extended with extra interaction terms between cell type and each of the K factors, such that,

$$\log \lambda_{ij} = \mu + a_i + b_j + \sum_{k=1}^{K} \eta_{ijk} + \varepsilon_{ij}$$
$$\eta_{ijk} = \begin{cases} \boldsymbol{z}_{ik}^\top \boldsymbol{u}_{jk} & \text{factor } k \text{ is a categorical variable with } L_k \text{ levels,} \\ \tilde{x}_{ik} u_{jk} & \text{factor } k \text{ is a numerical variable } (L_k = 1), \end{cases}$$

where η_{ijk} denotes the interaction effect between the cell type j and the factor k for the sample i, which is modelled by the interaction effect $\boldsymbol{u}_{jk} = (u_{jk1}, \ldots, u_{jkL_k})^\top$. Here $\tilde{x}_{ik}$ denotes the scaled value of x_{ik} (*i.e.*, sample mean and variance of the numerical factor k is 0 and 1) and $\boldsymbol{z}_{ik}^\top = (z_{ik1}, \ldots, z_{ikL_k})$ is a design vector whose element is

$$z_{jkl} = \begin{cases} 1 & x_{ik} = l, \\ 0 & \text{otherwise,} \end{cases}$$

for $l = 1, \ldots, L_k$. The interpretation of u_{jkl} is the log fold change of the jth cell type abundance for the lth level of categorical factor k against the grand mean. For a numerical factor k, u_{jk} is a scalar value reflecting the log fold change of the jth cell type abundance in response to one unit change of scaled data $\tilde{x}_{ik}$.

Because the factors are colinear and often confounding each other (unless the study is the designed experiment), we further assume those interaction effects follow multivariate normal distributions:

$$\boldsymbol{u}_{jk} \overset{i.i.d.}{\sim} \mathcal{N}(\boldsymbol{\mu}_k, \delta_k^2 I_{L_k}),$$

where $\boldsymbol{\mu}_k$ denotes the mean vector around which the variance parameter δ_k^2 is estimated, which reflects the relative contribution of each factor on cell type composition variation. Here the mean vector $\boldsymbol{\mu}_k$ is not the parameter of interest, therefore for the categorical factors, we regressed out from the model by assuming another multivariate normal distribution:

$$\boldsymbol{\mu}_k \overset{i.i.d.}{\sim} \mathcal{N}(0, \gamma_k^2 I_{L_k})$$

so that the number of parameters can be significantly reduced from L_k to 1.

Likelihood ratio test

To properly assess the statistical significance of each factor that explains a significant amount of interaction variation, we compared the the following two models:

$$H_0 : \delta_k^2 = 0$$
$$H_1 : \delta_k^2 > 0$$

Then the likelihood ratio test statistics follows the χ^2 distribution with one degree of freedom under the null hypothesis (H_0). In order to adjust multiple testing, we used the number of factors (*i.e.*, K, which is the same as the number of variance parameters γ_k^2 for cell type interaction) for the total number of tests.

Posterior mean and variance of random effects

In general, the generalised linear mixed model has no closed form of the marginal likelihood, because the integral with respect to random effects is intractable. Therefore an approximation becomes one of the natural alternatives. A well-known method of approximate integrals is named after Laplace (used in `lme4` package on `R`). Let $Y^\top = (Y_{11}, \ldots, Y_{NJ})$ be the vector of cell type counts and

$$\boldsymbol{u}^\top = (a_1, \ldots, a_N, b_1, \ldots, b_J, \boldsymbol{\mu}_1^\top, \ldots, \boldsymbol{\mu}_K^\top, \boldsymbol{u}_{11}^\top, \ldots, \boldsymbol{u}_{JK}^\top, \varepsilon_{11}, \ldots, \varepsilon_{NJ})$$

be the vector of all random effects, the marginal likelihood can be approximated as

$$p(Y) = \int p(Y|\boldsymbol{u})p(\boldsymbol{u})d\boldsymbol{u} \approx c|H|^{-\frac{1}{2}} \exp\{\mathcal{L}(\tilde{\boldsymbol{u}})\},$$

where $\mathcal{L}(\boldsymbol{u}) = \log p(Y|\boldsymbol{u})p(\boldsymbol{u})$ denotes the complete log likelihood function whose maximum is attained at $\boldsymbol{u} = \tilde{\boldsymbol{u}}$ with the first derivative $\mathcal{L}'(\tilde{\boldsymbol{u}}) = \boldsymbol{0}$ and the hessian matrix $H = -\mathcal{L}''(\tilde{\boldsymbol{u}})$, and c denotes a constant multiplication. This gives an approximated posterior distribution of $\boldsymbol{u}$ given Y, such that

$$\boldsymbol{u}|Y \sim \mathcal{N}(\tilde{\boldsymbol{u}}, H^{-1}).$$

Standard error of model parameters

The log marginal likelihood $\mathcal{L}(\boldsymbol{\theta}|Y) = \log p(Y)$ after integrating out the random effects $\boldsymbol{u}$ is a function of model parameters $\boldsymbol{\theta} = (\mu, \nu, \omega, \sigma, \gamma_1, \ldots, \gamma_K, \delta_1, \ldots, \delta_K)$. The standard error of $\boldsymbol{\theta}$ can be computed from the inverse matrix of the Fisher score matrix

$$\mathcal{I} = -\left.\frac{\partial^2 \mathcal{L}(\boldsymbol{\theta}|Y)}{\partial \boldsymbol{\theta} \partial \boldsymbol{\theta}^\top}\right|_{\boldsymbol{\theta}=\hat{\boldsymbol{\theta}}}$$

where the likelihood function attains its maximum value at $\boldsymbol{\theta} = \hat{\boldsymbol{\theta}}$ with $\mathcal{L}'(\hat{\boldsymbol{\theta}}|Y) = \mathbf{0}$.

Overdispersion due to technical variation

Although the Poisson model does not explicitly take account of the overdispersion in the cell type count data (unlike Negative Binomial distributions), the interaction term ε_{ij} between sample and cell type partly captures the discrepancy between $\mathbb{E}[Y_{ij}]$ and $\mathrm{Var}(Y_{ij})$, since

$$\mathrm{Var}(Y_{ij}|\boldsymbol{u}_{ij}) = \mathbb{E}[Y_{ij}|\boldsymbol{u}_{ij}] + \mathbb{E}[Y_{ij}|\boldsymbol{u}_{ij}]^2(e^{\sigma^2} - 1),$$

where $\boldsymbol{u}_{ij} = (a_i, b_j, \eta_{ij1}, \ldots, \eta_{ijK})^\top$. This fact suggests the model becomes overdispersed when $e^{\sigma^2} > 1$ given $\boldsymbol{u}_{ij}$.

Local true sign rate (*ltsr*)

To visualise how far each log fold change estimate $\tilde{u}$ deviates from 0 (in Mahalanobis distance), we computed the area under the curve of the posterior distribution with respect to u over the positive domain:

$$(ltsr) = \int_{u>0} \mathcal{N}(u|\tilde{u}, \sigma)du$$

if $\tilde{u} > 0$, where σ is obtained from the corresponding diagonal element of H^{-1}. Note that, if $\tilde{u} < 0$, the area under the curve over the negative domain is calculated.

Supplementary Note 2

Cell type enrichment analysis for GWAS traits

Bayesian hierarchical model and fGWAS

A Bayesian hierarchical model [2] has been proposed for mapping expression quantitative trait loci (eQTLs) with various functional annotations. The model is flexible and was extended later for fine-mapping and enrichment analysis of functional annotations for GWAS traits, called fGWAS [3]. The model has two prior probabilities, one is the variant-level prior probability and the other is the feature-level prior probability. The feature-level prior probability can be modelled as any functional annotation related with the feature. We used the cell type specific expression as the feature level annotation to quantify the relative enrichment of a cell type to a GWAS trait. The model is readily applicable to GWAS summary statistics without raw genotype and phenotype data.

Feature level prior probability and cell type enrichment

The cell type enrichment is measured by the effect size of cell type specific expression on GWAS associations. Let us denote by x_{jk} the expression level of the gene j $(j = 1, \dots, J)$ for the cell type k, and by Z_{jk} the Bernoulli random variable indicating the gene j for the cell type k is a putative causal gene for the GWAS trait, if $Z_{jk} = 1$; otherwise $Z_{jk} = 0$. We used the logistic regression model to estimate the cell type enrichment, such that

$$\text{logit}\, p(Z_{jk}) = \beta_{0k} + \beta_{1k} x_{jk},$$

where the effect size β_{1k} is the parameter of interest. Here $p(Z_{jk})$ is called the feature-level prior probability and Z_{jk} is unknown a priori (unobservable). We used a marginal genetic association around the gene j as a proxy of the fact that the gene j is causal to the GWAS trait. Let us denote by $p(y|Z_{jk})$ the conditional probability to observe GWAS phenotype y given Z_{jk}. The marginal probability is then given by

$$\begin{aligned} p(y) &= \prod_{j=1}^{J} [p(Z_{jk} = 0) p(y|Z_{jk} = 0) + p(Z_{jk} = 1) p(y|Z_{jk} = 1)] \\ &\propto \prod_{j=1}^{J} [(1 - \Pi_{jk}) + \Pi_{jk} RBF_j], \end{aligned}$$

where $\Pi_{jk} \equiv p(Z_{jk}) = \text{logit}^{-1}(\beta_{0k} + \beta_{1k} x_{jk})$ and

$$RBF_j = \frac{p(y|Z_{jk} = 1)}{p(y|Z_{jk} = 0)}$$

is so called the regional Bayes factor (RBF) to measure the strength of genetic association around the gene j. The detailed derivation of RBF_j is described in the next section.

The marginal probability is a function of $\beta = \{\beta_{0k}, \beta_{1k}\}$ and easily maximised using a standard EM algorithm (see Supplementary Note of [4]). The standard error of β_{1k} was estimated

from the inverse matrix of the Fisher information

$$\mathcal{I} = -\frac{\partial^2}{\partial\beta_k \partial\beta_k^\top} p(y)\bigg|_{\beta_k=\hat{\beta}_k}$$

at the maximum likelihood estimator $\hat{\beta}_k = \text{argmax}_{\beta_k} p(y)$. The P-value of cell type enrichment was calculated from the square of the Wald statistic, such that $\hat{\beta}_{1k}^2/\text{Var}(\hat{\beta}_{1k})$, which asymptotically follows the χ^2 distribution with 1 degree of freedom under the null hypothesis. For the multiple testing correction, we used the Benjamini-Hochberg method to compute Q-values across all cell types $(k = 1, \dots, K)$.

Regional Bayes factor

The regional Bayes factor was defined as a marginal genetic association averaged across all variants around the gene j. Let us denote a 1Mb cis-regulatory window $\mathcal{W}_j$ for gene j centred at the transcription start site (TSS). We aggregated the GWAS associations within the window as follows

$$RBF_j = \sum_{l \in \mathcal{W}_j} \pi_{jl} BF_{jl},$$

where π_{jl} is the variant-level prior probability that the genetic variant $l \in \mathcal{W}_j$ is the putative causal variant for the GWAS trait and BF_{jl} denotes the genetic association of the variant l to the GWAS trait. Here we used the Wakefield approximation [5] to convert the GWAS summary statistics, the effect size b_{jl} and its standard error s_{jl}, into

$$\begin{aligned}
\log BF_{jl} &= \frac{1}{2}\log(1 - r_{jl}) + \frac{z_{jl}^2}{2} r_{jl}, \\
z_{jl} &= \frac{b_{jl}}{s_{jl}}, \\
r_{jl} &= \frac{W}{W + s_{jl}^2}.
\end{aligned}$$

Here the prior variance of the effect size was set to be $W = 0.1$ [3].

It is noticeable that the window $\mathcal{W}_j$ may overlap each other in a gene dense region and therefore the variant $l \in \mathcal{W}_j$ could appear multiple times in other windows. This may lead to the underestimation of the standard error of β. To overcome this issue, we hypothesised that 60% of interactions is observed in 20Kb distance or less, because the cis-regulatory interaction between a putative causal variant and a gene promoter occurred in a very short distance [4]. The distribution can be approximated by the exponential distribution with the rate parameter equal to $\hat{\lambda} \approx 4.58 \times 10^{-5}$. Then the variant-level prior probability that the variant $l \in \mathcal{W}_j$ is a putative causal variant given TSS proximity $d_{jl} \in [0, 500Kb]$ is obtained by

$$\pi_{jl} = \frac{e^{-\hat{\lambda} d_{jl}}}{\sum_{m \in \mathcal{W}_j} e^{-\hat{\lambda} d_{jm}}}.$$

This prior probability strongly penalised the long-range association from the TSS and therefore the overlapping effect is potentially minimised.

Linkage disequilibrium

Linkage disequilibrium (LD) could potentially bias the marginal association. We computed the unbiased LD score [6], w_{jl} ($l \in \mathcal{W}_j$) inside the window to properly weight the variant-level prior probability, such that

$$\tilde{\pi}_{jl} = \frac{w_{jl}^{-1} e^{-\hat{\lambda} d_{jl}}}{\sum_{m \in \mathcal{W}_j} w_{jm}^{-1} e^{-\hat{\lambda} d_{jm}}}.$$

Intuitively, if the variant l has a lot of LD mates, we down-weight the association Bayes factor by $1/w_{jl}$.

Adjustment by marginal expression

In reality, cell type specific expressions are strongly correlated between different cell types and we observe large effect size $\hat{\beta}_{1k}$ even if the cell type k is not relevant for the GWAS trait [7]. We introduced the marginal expression $\bar{x}_j = \sum_{k=1}^{K} x_{jk}/K$ for the gene j across K different cell types in the data to adjust cell type enrichment in the model, such that

$$\text{logit}\, p(Z_{jk}) = \beta_{0k} + \beta_{1k} x_{jk} + \beta_{2k} \bar{x}_j.$$

Again, β_{1k} is the parameter of interest and the parameters $\{\beta_{0k}, \beta_{1k}, \beta_{2k}\}$ are easily maximised using a standard EM algorithm (see Supplementary Note of [4]).

Supplementary Note 3

Description of tuft cell activation results related to the Extended Data Figure 7

*PLCG*2 expression by tuft cells was at higher levels than B and myeloid cell lineages (Extended Data Fig. 7a) and together with downstream signaling mediators including *RAC*2, *ITPR*2, *PRKCA* and *TRPM*5 suggested the ability of tuft cells to respond to immune cells. In addition, we observe an increase in PLCG2 expression across TNF-alpha or IFN-gamma stimulated human organoid epithelial cells (Extended Data Fig. 7d-e) and show a significant increase in inhibitory FcγRIIb-expressing tuft cells in a mouse model of intestinal colitis, suggesting a negative feedback mechanism at play during chronic inflammation (Extended Data Fig. 7f). This data suggests the likely capacity of tuft cells to sense IgG through PLCG2 activation.

Supplementary Note 4

Description of B cell gene expression and BCR analysis results related to the Extended Data Figure 14

To infer whether the developing lymphoid structures were partitioned into B and T cells zones, we characterised B cells in fetal and adult tissues (Extended Data Fig. 14a-g). Analysis of paired V(D)J sequencing data revealed almost exclusive IgM heavy chain expression across all fetal gut B cells and no meaningful levels of clonal expansion and somatic mutation (Extended Data Fig. 14h-i). In comparison, adult B cells expressed primarily IgA1 and IgA2 and displayed high mutational frequency and clonal expansion consistent with having undergone affinity maturation (Extended Data Fig. 14h-i). Mutation frequency and clonal expansion were slightly higher at the proximal and distal ends of the adult gut, and sharing of B cell clones, while restricted to occurring within each donor, was evident across distant gut regions (Extended Data Fig. 14j-l). This suggests that additional environmental factors, such as microbiota, might be required for the maturation and zonation of B cells in developing secondary lymphoid organs.

Supplementary References

[1] Agresti A (2018) An Introduction to Categorical Data Analysis. John Wiley & Sons.

[2] Veyrieras JB, Kudaravalli S, Kim SY, Dermitzakis ET, Gilad Y, et al. (2008) High-resolution mapping of expression-QTLs yields insight into human gene regulation. PLoS Genet 4: e1000214.

[3] Pickrell JK (2014) Joint analysis of functional genomic data and genome-wide association studies of 18 human traits. Am J Hum Genet 94: 559–573.

[4] Kumasaka N, Knights AJ, Gaffney DJ (2019) High-resolution genetic mapping of putative causal interactions between regions of open chromatin. Nat Genet 51: 128–137.

[5] Wakefield J (2009) Bayes factors for genome-wide association studies: comparison with p-values. Genet Epidemiol 33: 79–86.

[6] Bulik-Sullivan BK, Loh PR, Finucane HK, Ripke S, Yang J, et al. (2015) LD score regression distinguishes confounding from polygenicity in genome-wide association studies. Nat Genet 47: 291–295.

[7] Watanabe K, Umićević Mirkov M, de Leeuw CA, van den Heuvel MP, Posthuma D (2019) Genetic mapping of cell type specificity for complex traits. Nat Commun 10: 3222.
