# Supplementary material for: Cells of the human intestinal tract mapped across space and time
Source: Nature. 2021 Sep 8;597(7875):250–5. doi: 10.1038/s41586-021-03852-1 (PMC8426186; doi:10.1038/s41586-021-03852-1)
Supplement: Supplementary file 2 — Reporting Summary [file 41586_2021_3852_MOESM2_ESM.pdf]

nature research

Corresponding author(s): Kylie James and Sarah A. Teichmann

Last updated by author(s): Jun 1, 2021

# Reporting Summary

Nature Research wishes to improve the reproducibility of the work that we publish. This form provides structure for consistency and transparency in reporting. For further information on Nature Research policies, see our Editorial Policies and the Editorial Policy Checklist.

## Statistics

For all statistical analyses, confirm that the following items are present in the figure legend, table legend, main text, or Methods section.

| n/a | Confirmed | |
|---|---|---|
| ☐ | ☒ | The exact sample size ($n$) for each experimental group/condition, given as a discrete number and unit of measurement |
| ☐ | ☒ | A statement on whether measurements were taken from distinct samples or whether the same sample was measured repeatedly |
| ☐ | ☒ | The statistical test(s) used AND whether they are one- or two-sided<br>*Only common tests should be described solely by name; describe more complex techniques in the Methods section.* |
| ☐ | ☒ | A description of all covariates tested |
| ☐ | ☒ | A description of any assumptions or corrections, such as tests of normality and adjustment for multiple comparisons |
| ☐ | ☒ | A full description of the statistical parameters including central tendency (e.g. means) or other basic estimates (e.g. regression coefficient) AND variation (e.g. standard deviation) or associated estimates of uncertainty (e.g. confidence intervals) |
| ☐ | ☒ | For null hypothesis testing, the test statistic (e.g. $F$, $t$, $r$) with confidence intervals, effect sizes, degrees of freedom and $P$ value noted<br>*Give P values as exact values whenever suitable.* |
| ☒ | ☐ | For Bayesian analysis, information on the choice of priors and Markov chain Monte Carlo settings |
| ☐ | ☒ | For hierarchical and complex designs, identification of the appropriate level for tests and full reporting of outcomes |
| ☐ | ☒ | Estimates of effect sizes (e.g. Cohen's $d$, Pearson's $r$), indicating how they were calculated |

*Our web collection on statistics for biologists contains articles on many of the points above.*

## Software and code

Policy information about availability of computer code

| | |
|---|---|
| Data collection | Software used include: 10x Genomics CellRanger (v3.0.0, v3.0.2) , 10x Genomics vdj (v.3.1.0 ), 10x Genomics SpaceRanger (v1.2.1 ), STAR aligner (Smart-seq2 data; version 2.5.1b). Visium spatial transcriptomics samples were aligned using 10x Genomics SpaceRanger (v1.2.1 ). 10X TCR sequences were aligned using 10x Genomics vdj (v.3.1.0 ) and Smartseq2 T cell receptor sequences were determined using TraCeR software (https://hub.docker.com/r/teichlab/tracer/). |
| Data analysis | Single cell data analysis was performed using Python (version 3), R (3.5.3), Pandas (version 0.24.2), limma (v3.46.0), NumPy (version 0.25.2), Anndata (version 0.6.19), Seaborn (version 0.11.0), and ScanPy (version 1.4 and 1.5.1). Ambient mRNA was removed using SoupX_1.4.8. Doublet removal using Scrublet (version 0.2.1). Batch correction- bbknn (version 1.3.9). TIgGER (v.03.1) , ChangeO (v.0.4.5), Shazam (v.0.1.11) were used for BCR analysis. Pseudotime calculated using scVelo (0.21) and Scanpy (1.5.1). CellPhoneDB (v2.0) was used for ligand-receptor analysis. Cell type enrichment analysis performed using miloR (https://github.com/MarioniLab/miloR). Flow cytometry data was visualised using FlowJo software (Version 10.7.0, Tree Star Inc.).GraphPadPrism 7 software was used for analysing FACS data. Additional custom codes used in this manuscript are available at Github: https://github.com/Teichlab/SpaceTimeGut, https://github.com/vitkl/fetal_gut_mapping/, https://github.com/natsuhiko/PHM. |

For manuscripts utilizing custom algorithms or software that are central to the research but not yet described in published literature, software must be made available to editors and reviewers. We strongly encourage code deposition in a community repository (e.g. GitHub). See the Nature Research guidelines for submitting code & software for further information.

## Data

Policy information about availability of data

All manuscripts must include a data availability statement. This statement should provide the following information, where applicable:

- Accession codes, unique identifiers, or web links for publicly available datasets
- A list of figures that have associated raw data
- A description of any restrictions on data availability

The expression data for fetal and adult regions is available in an interactive browsing website: www.gutcellatlas.org. Raw sequencing data are available at ArrayExpress (www.ebi.ac.uk/arrayexpress/; accession numbers E-MTAB-9543, E-MTAB-9536, E-MTAB-9532, E-MTAB-9533 and E-MTAB-10386).

# Field-specific reporting

Please select the one below that is the best fit for your research. If you are not sure, read the appropriate sections before making your selection.

☒ Life sciences ☐ Behavioural & social sciences ☐ Ecological, evolutionary & environmental sciences

For a reference copy of the document with all sections, see nature.com/documents/nr-reporting-summary-flat.pdf

# Life sciences study design

All studies must disclose on these points even when the disclosure is negative.

| | |
|---|---|
| Sample size | Sample size were determined by availability of donors within the sampling time-frame. No statistical methods were used to calculate appropriate sample size. We followed standards in the field and Human Cell Atlas criteria. |
| Data exclusions | No exclusion was applied to the uploaded raw data in ArrayExpress. For the final count matrix, we excluded cells based on pre-established criteria for single cells. We excluded doublets and ow quality cells. This criteria is further summarised in the Methods section. |
| Replication | Single-cell RNA sequencing was carried at on gut and lymph node tissue from 7 fetal donors and 4 adult diseased organ donors. All technical and biological replications of experiments were successful. |
| Randomization | Sample collection was based on availability of fetal and warm-autopsy donors. Since we were following developmental stage of the intestinal tract, we allocated donor samples into developmental groups based on age. |
| Blinding | This study made no comparison between discreet groups for human participants, thus binding of investigators was not necessary. For mouse studies, Tuft and non-tuft epithelial cells from the same mice were analysed, thus binding was not relevant. |

# Reporting for specific materials, systems and methods

We require information from authors about some types of materials, experimental systems and methods used in many studies. Here, indicate whether each material, system or method listed is relevant to your study. If you are not sure if a list item applies to your research, read the appropriate section before selecting a response.

### Materials & experimental systems

| n/a | Involved in the study | |
|---|---|---|
| ☐ | ☒ | Antibodies |
| ☒ | ☐ | Eukaryotic cell lines |
| ☒ | ☐ | Palaeontology and archaeology |
| ☐ | ☒ | Animals and other organisms |
| ☐ | ☒ | Human research participants |
| ☒ | ☐ | Clinical data |
| ☒ | ☐ | Dual use research of concern |

### Methods

| n/a | Involved in the study | |
|---|---|---|
| ☒ | ☐ | ChIP-seq |
| ☐ | ☒ | Flow cytometry |
| ☒ | ☐ | MRI-based neuroimaging |

## Antibodies

| | |
|---|---|
| Antibodies used | EpCAM-FITC (1:400, G8.8, Invitrogen, cat: 11-5791-82), anti-mouse CD45-Bv650 (1:200, 30-F11, BioLegend, cat: 103151), CD11b-Bv421 (1:300, M1/70, BD Biosciences, cat: 562632), Siglec- F-APC (1:200, 1RNM44N, Invitrogen, cat: 12-1702-82), FcγRI-PE (1:200, X54-5/7.1, BioLegend, cat: 139303), FcγRIIB-PE (1:200, AT130-2, Invitrogen, cat: 12-0321-82), FcγRII/RIII-PE (1:200, 2.4G2, BD Biosciences, cat: 553145), FcγRIV-PE (1:200, 9E9, BioLegend, cat: 149503) and Rat IgG2b, κ isotype-PE-Cy7 (1:200, LOU/C, BD Biosciences, cat: 552849), Brilliant Violet 650 mouse anti-human CD45 (dilution: 1:200, Biolegend, cat: 304043), Alexa Fluor 700 mouse anti-human CD4 (dilution: 1:200, Biolegend, cat: 300526), and APC-H7 mouse anti-human CD19 (dilution: 1:200, BD biosciences, cat: 560727). |

| | |
|---|---|
| Validation | EpCAM-FITC: Validated against TE-71 cell line compared to isotype control (Rat IgG2a K Isotype Control FITC). FACS plot shown on website.<br>anti-mouse CD45-Bv650: Staining of C57BL/6 mouse splenocytes. FACS plot shown on website.<br>CD11b-Bv421: Provided positive staining of C57BL/6 mouse bone marrow cells. FACS plot shown on website.<br>Siglec- F-APC: Validated as staining of mouse thioglycolate-elicited peritoneal exudate cells. FACS plot shown on website.<br>FcγRIIB-PE: positive staining of mouse splenocytes and compared to an isotype control. FACS plot shown on website.<br>FcγRI-PE: Validated on C57BL/6 bone marrow cells and compared to isotype control (Armenian hamster IgG PE). FACS plot shown on website.<br>FcγRII/RIII-PE: Validated by flow cytometric analysis of CD16/CD32 expression on mouse splenocytes. FACS plot shown on website.<br>FcγRIV-PE: Positive staining of C57BL/6 bone marrow cells compared to isotype control (Armenian hamster IgG PE). FACS plot shown on website.<br>Rat IgG2b, κ isotype-PE-Cy7: isotype control.<br>Brilliant Violet 650 mouse anti-human CD45: Validated in human peripheral blood lymphocytes. FACS plot shown on website.<br>Alexa Fluor 700 mouse anti-human CD4: Routinely tested and used for human peripheral blood lymphocytes. FACS plot shown on website.<br>APC-H7 mouse anti-human CD19: Validated for CD19 expression on human lysed whole blood. FACS plot shown on website. |

## Animals and other organisms

Policy information about studies involving animals; ARRIVE guidelines recommended for reporting animal research

| | |
|---|---|
| Laboratory animals | C57BL/6 mice were obtained from Jackson Laboratories (Margate, UK) and housed in specific pathogen-free conditions at a Home Office-approved facility at the University of Cambridge. Mice were maintained with a 12 hour light/ 12 hour dark cycle, with temperature ranging from 20-24°C and humidity of 45-65%. Female mice aged 10-14 weeks were used in experiments. |
| Wild animals | No wild animals were used in the study. |
| Field-collected samples | No field collected samples were used in the study. |
| Ethics oversight | All procedures were carried out in accordance with ethical guidelines with the United Kingdom Animals (Scientific Procedures) Act of 1986 and approved by The University of Cambridge Animal Welfare and Ethical Review Body. |

Note that full information on the approval of the study protocol must also be provided in the manuscript.

## Human research participants

Policy information about studies involving human research participants

| | |
|---|---|
| Population characteristics | Human fetal gut samples were obtained from the Human Developmental Biology Resource (HDBR, www.hdbr.org) and age ranged between 7-17 post-conception weeks.<br>Organ donors were either male or female and age ranged from 20-75 years. Ethnicity was not recorded, but expected to be primary Caucasian.<br>Pediatric (5-12 years; either male or female) patient biopsy material used in intestinal organoid culture. |
| Recruitment | Pediatric patient material used in intestinal organoid culture was obtained with informed consent from either parents and/or patients using age appropriate consent and assent forms as part of the ethically approved research study (REC-96/085).<br><br>Human adult tissue was obtained by the Cambridge Biorepository of Translational Medicine from deceased transplant organ donors after ethical approval (reference 15/EE/0152, East of England—Cambridge South Research Ethics Committee) and informed consent from the donor families.<br><br>Human fetal gut samples were obtained from the Human Developmental Biology Resource (HDBR, www.hdbr.org). The maternal consent was obtained through Newcastle hospital or through Cambridge Addenbrooke's hospital in collaboration with Roger A. Barker. |
| Ethics oversight | Procurement and study of fetal samples were approved by REC18/NE/0290- IRAS project ID: 250012, North East - Newcastle & North Tyneside 1 Research Ethics Committee and REC-96/085, East of England - Cambridge Central Research Ethics Committee.<br><br>Pediatric patient material used in intestinal organoid culture was obtained with informed consent from either parents and/or patients using age appropriate consent and assent forms as part of the ethically approved research study (REC-12/EE/0482, NRES Committee East of England, Hertfordshire and REC-17/EE/0265- IRAS project ID: 222907, East of England - Cambridge South Research Ethics Committee).<br><br>Human adult tissue was obtained by the Cambridge Biorepository of Translational Medicine from deceased transplant organ donors after ethical approval (reference 15/EE/0152, East of England—Cambridge South Research Ethics Committee) and informed consent from the donor families. |

Note that full information on the approval of the study protocol must also be provided in the manuscript.

## Flow Cytometry

### Plots

Confirm that:

☒ The axis labels state the marker and fluorochrome used (e.g. CD4-FITC).

☒ The axis scales are clearly visible. Include numbers along axes only for bottom left plot of group (a 'group' is an analysis of identical markers).

☒ All plots are contour plots with outliers or pseudocolor plots.

☒ A numerical value for number of cells or percentage (with statistics) is provided.

### Methodology

| | |
|---|---|
| Sample preparation | C57BL/6 mice received either normal drinking water or 2% (w/v) 36,000-50,000MW dextran sodium sulfate (DSS) (MP Biomedicals) to induce DSS colitis. For DSS treatment, mice received DSS water for 5 days followed by 14 days of normal drinking water, and then a final 5 days of 2% (w/v) DSS prior to being culled.<br>Small intestines of mice were flushed of faecal content with ice-cold PBS, opened longitudinally, cut into 0.5 cm pieces, and washed by vortexing three times with PBS with 10mM HEPES. Tissue was then incubated with an epithelial stripping solution (RPMI-1640 with 2% (v/v) FCS, 10mM HEPES, 1mM DTT, and 5 mM EDTA) at 37°C for two intervals of 20 minutes to remove epithelial cells. The epithelial fraction was subsequently incubated at 37°C for 10 minutes with dispase (0.3 U/mL, Sigma-Aldrich) and passed through a 100μm filter to obtain a single-cell suspension. Cells were blocked for 20 minutes at 4°C with 0.5% (v/v) heat-inactivated mouse/rat serum followed by extracellular staining in PBS at 4°C for 45 minutes with the following antibodies; EpCAM-FITC (1:400, G8.8, Invitrogen), CD45-Bv650 (1:200, 30-F11, BioLegend), CD11b-Bv421 (1:300, M1/70, BD Biosciences), Siglec-F-APC (1:200, 1RNM44N, Invitrogen), FcγRI-PE (1:200, X54-5/7.1, BioLegend), FcγRIIB-PE (1:200, AT130-2, Invitrogen), FcγRII/RIII-PE-Cy7 (1:200, 2.4G2, BD Biosciences), FcγRIV-PE (1:200, 9E9, BioLegend) and Rat IgG2b, κ isotype-PECy7 (1:200, LOU/C, BD Biosciences). Cells were then stained with LIVE/DEAD Fixable Aqua Dead Cell Stain Kit (Thermo Fisher Scientific) for 20 minutes at room temperature, fixed with 2% PFA, and analysed on a CytoFLEX LX (Beckman Coulter) flow cytometer. |
| Instrument | CytoFLEX LX (Beckman Coulter) flow cytometer. |
| Software | Flow cytometry data was analyzed using FlowJo software (Version 10.7.0, Tree Star Inc.) |
| Cell population abundance | EpCAM+SiglecF- cells that were defined as non-tuft epithelial cells were approximately 85% of live cells as analysed by flow cytometry. EpCAM+SiglecF+ cells made up 4%. This latter population was further assessed for CD11b cells in order to remove possible myeloid cells. Tuft cells from this gating were defined as EpCAM+SiglecF+CD11b- cells and represented approximately 94%. The purity of these populations was not determined as these cells were not sorted after analysis.<br><br>These proportions are included in the gating strategy for these populations in Extended data 4j. |
| Gating strategy | Mouse tuft cells were defined as EpCAM+SiglecF+CD11b- cells in epithelial fractions and non-tuft epithelial cells were defined as EpCAM+SiglecF-CD11b- cells in epithelial fractions. |

☒ Tick this box to confirm that a figure exemplifying the gating strategy is provided in the Supplementary Information.
